# H-CRRETAWAC-OH, a Lead Structure for the Development of Radiotracer Targeting Integrin *α*_**5**_ *β*_**1**_?

**DOI:** 10.1155/2014/243185

**Published:** 2014-10-13

**Authors:** Roland Haubner, Simone Maschauer, Jürgen Einsiedel, Iris E. Eder, Christine Rangger, Peter Gmeiner, Irene J. Virgolini, Olaf Prante

**Affiliations:** ^1^Department of Nuclear Medicine, Innsbruck Medical University, Anichstraße 35, 6020 Innsbruck, Austria; ^2^Department of Nuclear Medicine, Molecular Imaging and Radiochemistry, Friedrich-Alexander University Erlangen-Nürnberg (FAU), Schwabachanlage 6, 91054 Erlangen, Germany; ^3^Department of Chemistry and Pharmacy, Emil Fischer Center, Friedrich-Alexander University Erlangen-Nürnberg (FAU), Schuhstraße 19, 91052 Erlangen, Germany; ^4^Department of Urology, Innsbruck Medical University, Anichstraße 35, 6020 Innsbruck, Austria

## Abstract

Imaging of angiogenic processes is of great interest in preclinical research as well as in clinical settings. The most commonly addressed target structure for imaging angiogenesis is the integrin *α*_v_ *β*_3_. Here we describe the synthesis and evaluation of [^18^F]FProp-Cys^*^-Arg-Arg-Glu-Thr-Ala-Trp-Ala-Cys^*^-OH, a radiolabelled peptide designed to selectively target the integrin *α*_5_ *β*_1_. Conjugation of 4-nitrophenyl-(*RS*)-2-[^18^F]fluoropropionate provided [^18^F]FProp-Cys^*^-Arg-Arg-Glu-Thr-Ala-Trp-Ala-Cys^*^-OH in high radiochemical purity (>95%) and a radiochemical yield of approx. 55%. In vitro evaluation showed *α*_5_ *β*_1_ binding affinity in the nanomolar range, whereas affinity to *α*_v_ *β*_3_ and *α*_IIb_ *β*_3_ was >50 *μ*M. Cell uptake studies using human melanoma M21 (*α*_v_ *β*_3_-positive and*α*_5_ *β*_1_-negative), human melanoma M21-L (*α*_v_ *β*_3_-negative and *α*_5_ *β*_1_-negative), and human prostate carcinoma DU145 (*α*_v_ *β*_3_-negative and *α*_5_ *β*_1_-positive) confirmed receptor-specific binding. The radiotracer was stable in human serum and showed low protein binding. Biodistribution studies showed tumour uptake ranging from 2.5 to 3.5% ID/g between 30 and 120 min post-injection. However, blocking studies and studies using mice bearing *α*_5_ *β*_1_-negative M21 tumours did not confirm receptor-specific uptake of [^18^F]FProp-Cys^*^-Arg-Arg-Glu-Thr-Ala-Trp-Ala-Cys^*^-OH, although this radiopeptide revealed high affinity and substantial selectivity to *α*_5_ *β*_1_ in vitro. Further experiments are needed to study the in vivo metabolism of this peptide and to develop improved radiopeptide candidates suitable for PET imaging of *α*_5_ *β*_1_ expression in vivo.

## 1. Introduction

Angiogenesis is a critical step in the formation of cancer. A variety of molecular processes are involved in the formation of new blood vessels out of the preexisting vasculature. Numerous therapeutic strategies in oncology are focused on the inhibition of tumour-induced angiogenesis [1–3]. These include approaches to inhibit VEGF, MMP, or integrin interactions. Thus, there is great interest in techniques which allow noninvasive monitoring of molecular target structures involved in these angiogenic processes [4]. One of the most prominent target structures used for the development of radiopharmaceuticals for imaging angiogenesis yet is the integrin*α*_*v*_ *β*_3_ [5]. It has been shown that this integrin is involved in endothelial cell/matrix interactions during tumour-induced formation of new vessels as well as in the mediation of tumour cell migration during invasion and extravasation [6]. A series of studies using a variety of different radiopharmaceuticals have already demonstrated that noninvasive determination of*α*_*v*_ *β*_3_ expression is feasible [5, 7]. This opens the possibility to use single photon emission tomography (SPET) as well as positron emission tomography (PET) techniques to support information for planning and controlling corresponding antiangiogenic therapies.

In contrast to the data found in a variety of inhibition studies, suggesting a critical role of*α*_*v*_ *β*_3_ during angiogenesis and which were the basis for the development of antagonists for this receptor, genetic studies indicate that the integrin*α*_*v*_ *β*_3_ is not required for angiogenesis [8]. An explanation for this discrepancy could be found in studies demonstrating that animals lacking*α*_*v*_ *β*_3_ develop compensatory changes in VEGF signalling, which permit angiogenesis to occur during embryogenesis [9]. Anyway, genetic ablation of the integrin*α*_5_ *β*_1_, the major fibronectin-binding integrin, leads to severe vascular abnormalities [10] indicating that this integrin may play an even more important role than the integrin*α*_*v*_ *β*_3_ in neovascularisation. Additionally, this integrin is upregulated in tumour blood vessels and plays a role in tumour angiogenesis and tumour growth [11, 12]. Based on these findings, the integrin*α*_5_ *β*_1_ has become another target structure in the development of radiopharmaceuticals for imaging angiogenesis.

Recently, nonpeptide antagonists for the integrin*α*_5_ *β*_1_ based on an aza-glycine scaffold with low nanomolar affinity for*α*_5_ *β*_1_ and up to 10^4^-fold higher selectivity when compared with*α*_*v*_ *β*_3_ have been developed [13]. Subsequently, the most promising aza-glycine derivative was modified by conjugation with the ^68^Ga-chelator NODAGA [14].* In vitro* experiments using a competitive solid phase integrin binding assay have demonstrated receptor specific binding, and* in vivo* studies with the ^68^Ga-labelled derivative have indicated the feasibility to visualize integrin*α*_5_ *β*_1_-expression in murine tumour models using small animal PET [14].

Our approach to develop a radiopharmaceutical for the noninvasive determination of integrin*α*_5_ *β*_1_-expression is based on a disulfide bridged cyclic peptide H-Cys^*^-Arg-Arg-Glu-Thr-Ala-Trp-Ala-Cys^*^-OH (H-C^*^RRETAWAC^*^-OH; asterisks indicate a disulfide bridge) [15] resulting from a phage display library. It was demonstrated that this compound inhibits the binding of fibronectin to*α*_5_ *β*_1_ (IC_50_ of 8 nM) and the attachment of*α*_5_ *β*_1_-positive cells. In this work, we describe the synthesis of H-C^*^RRETAWAC^*^-OH, its binding affinity for the integrins*α*_*v*_ *β*_3_,*α*_5_ *β*_1_, and*α*_*IIb*_ *β*_3_, and the radiolabelling of the peptide via [^18^F]fluoropropionic acid. After careful verification of the*α*_5_ *β*_1_ expression in the cells lines used in this study by FACS analysis, we applied* in vitro* assays for cell uptake studies and the determination of tracer stability in serum. Finally, the biodistribution of [^18^F]FProp-CRRETAWAC-OH was studied to assess the applicability of the radiopeptide for imaging of*α*_5_ *β*_1_ expression* in vivo* by small animal PET.

## 2. Materials and Methods

All reagents were used as supplied without further purification. 9-Fluorenylmethoxycarbonyl (Fmoc) protected amino acids and tritylchloride polystyrene resins (TCP resin; H-Cys-2-ClTrt resin) were purchased from Novabiochem of Merck Millipore Group (Darmstadt, Germany). The coupling reagents 1-hydroxy-7-azabenzotriazole (HOAt) and* O*-(7-azabenzotriazol-1-yl)-1,1,3,3-tetramethyl uroniumhexafluorophosphate (HATU) were purchased from GenScript Corporation (Piscataway, NJ). All other organic reagents were obtained from VWR International GmbH (Darmstadt, Germany) or Sigma-Aldrich Handels GmbH (Buchs, Switzerland). [^18^F]fluoride was purchased from IASON GmbH (Graz, Austria) and supplied fixed on a cartridge.

Human melanoma M21 and M21-L cells were a kind gift from D. A. Cheresh, Departments of Immunology and Vascular Biology, The Scripps Research Institute, La Jolla, CA. DU145 human prostate cancer cells were obtained from ATCC (Rockville, MD, USA).

Liquid chromatography mass spectrometry (LC-MS) analysis was carried out using a PepMap C18 column (150 mm × 1 mm ID; 3 *μ*m particle size; ICT, Vienna, Austria). The LC system was directly coupled to a Finnigan MAT LCQ ion trap instrument (San Jose, CA) equipped with an electrospray source. Samples were analysed by HPLC (127 Solvent Module, Beckman Instruments, Palo Alto, CA).

NMR spectra were measured with a Bruker AM 360 (Bruker, Karlsruhe, Germany) at 300 K. NMR chemical shifts are reported in ppm relative to trimethylsilane.

The radioactivity of the samples was measured using a 2480 Automatic Gamma Counter Wizard^2^ 3′′ (Perkin Elmer, Vienna, Austria).

### 2.1. Synthesis

#### 2.1.1. Sodium (*RS*)-2-Fluoropropionate (FProp)

The synthesis is based on the procedure described in [16]. Briefly, a mixture of potassium fluoride (55 g, 0.95 mol; dried at 150°C for 3 h), acetamide (50 g; dried for 7 days over phosphor pentoxide), and 2-bromopropionic acid ethyl ester (33 g, 0.18 mol) was stirred for 3 h at 100°C. After cooling to room temperature, the mixture was dissolved in water and the organic compounds were extracted with diethyl ether. The ether was removed in vacuo and the product was isolated via vacuum distillation (220 mbar, 72–75°C; 12 g, 0.1 mol; yield 55%).

For hydrolysis, the ester was heated under reflux with an equimolar amount of sodium hydroxide in water/methanol (1 : 1) for 30 min. Subsequently, the solvent was removed in vacuo. ^1^H NMR (360 MHz, DMSO-*d*_6_) *δ* 1.32 (dd, *J* = 23.3, 6.7 Hz, 3H, CH_3_), 4.55 (dd, *J* = 52.5, 6.7 Hz, 1H, CFH).

#### 2.1.2. H-C ^*^RRETAWAC ^*^-OH

Synthesis of the linear peptide H-CRRETAWAC-OH followed standard solid phase peptide synthesis (SPPS) protocols using Fmoc-protection group strategy and was carried out according to the procedure described in [17]. Disulfide bridge formation was performed in analogy to Schottelius et al. [18]. Briefly, the peptide (100 mg) was suspended in tetrahydrofuran (15 mL) and ammonium acetate (5 mM) was added until the formation of a clear solution. The solution was adjusted to pH 7.0 by dropwise addition of sodium bicarbonate, and, subsequently, 30% hydrogen peroxide (35 *μ*L) was added. After stirring for 120 min at room temperature, the solution was removed by evaporation, the residue was dissolved in dimethylformamide (DMF), and the product was precipitated by the addition of diethyl ether. The suspension was centrifuged and the precipitate washed with diethyl ether.

The isolation of H-C^*^RRETAWAC^*^-OH via semipreparative RP-HPLC was performed using a Gilson 322 HPLC pump with a Gilson UV/VIS-155 detector (Gilson International B.V., Limburg, Germany) and a MultoHigh 100 RP 18 5 *μ*m, 250 × 10 mm column (CS—Chromatographie Service GmbH, Langerwehe, Germany). Flow rate was 5 mL/min. For preparation, corresponding CH_3_CN/H_2_O/0.1% TFA gradients were used.

Peptide purity and identity were assessed by analytical HPLC (Dionex P680 HPLC pump, Dionex UVD 170 U UV/VIS detector (Dionex, Germering, Germany), Bioscan radiometric detector (Bioscan, Washington, DC), and a Bischoff Nucleosil 120-5 C18 250 × 4.6 mm column (Leonberg, Germany); flow rate 1 mL/min; UV detection at 220 nm) employing the following eluent system: 5–65% CH_3_CN/0.1% TFA in 30 min, purity: >95% (*t*_*r*_: 13.0 min) and ESI-MS: calcd for C_44_H_68_N_16_O_13_S_2_: 1092.6, found:* m/z* 1093.5 [M+H]^+^.

#### 2.1.3. FProp-C^*^RRETAWAC^*^-OH

For production of FProp-C^*^RRETAWAC^*^-OH, microwave assisted (discover microwave oven, CEM Corp., Kamp-Lintfort, Germany) peptide synthesis was carried out in glass tubes loosely sealed with a silicon septum. To avoid overpressure, DMF was used as solvent. After each irradiation step, intermittent cooling of the reaction mixture to a temperature of −10°C was achieved by sufficient agitation in an ethanol-ice bath. Preparative RP-HPLC was performed using Agilent (Waldbronn, Germany) 1100 preparative series (column: Zorbax Eclipse XDB-C8, 21.2 mm × 150 mm, 5 *μ*m particles, flow rate: 10 mL/min, detection wavelength: 220 nm) and solvent systems as specified below. Purity and identity were assessed by analytical RP-HPLC (Agilent 1100 analytical series, column: Zorbax Eclipse XDB-C8 analytical column, 4.6 mm × 150 mm, 5 *μ*m, flow rate: 0.5 mL/min, detection wavelength: 220 nm) coupled to a Bruker Esquire 2000 mass detector (Bruker Daltonics, Bremen, Germany) equipped with an ESI-trap. The solvent system is specified below. ESI-TOF high mass accuracy and resolution experiments were performed on a BRUKER maXis MS (Bruker Daltonics, Bremen) in the laboratories of the Chair of Organic Chemistry (Professor Dr. Rik Tykwinski), Department of Pharmacy and Chemistry, Friedrich-Alexander University Erlangen-Nürnberg (FAU).

The peptide synthesis was achieved starting from H-Cys-2-ClTrt resin (100 mg, loading 0.56 mmol/g). The amino acids were incorporated as their commercially available derivatives in the following order: Fmoc-Ala-OH, Fmoc-Trp(Boc)-OH, Fmoc-Thr(*t*Bu)-OH, Fmoc-Glu(O*t*Bu)-OH, and Fmoc-Arg(Pbf)-OH. Elongation of the peptide chain was done by repetitive cycles of Fmoc-deprotection applying 20% piperidine in DMF (microwave irradiation: 5 × 5 s, 100 W), followed by 5 washing steps with DMF and subsequent peptide coupling using the following conditions: amino acid/PyBOP/diisopropylethylamine HOBt (5 eq/5 eq/5 eq/7.5 eq). Exception was made for Fmoc-Trp(Boc)-OH, which (5 eq) was coupled for a second time with HATU/DIPEA (5 eq/5 eq). The last cysteine residue was introduced as the pentafluorophenyl ester (Fmoc-Cys(Trt)-OPfp, 5 eq) without addition of base. Coupling of sodium (*RS*)-2-fluoropropionate (5 eq) was carried out using HATU/DIPEA (5 eq/7 eq) activation. All building blocks and reagents were dissolved in a minimum amount of DMF and microwave irradiation was performed 15 × 10 s employing 50 W. After the last acylation step, the resin was 10 times rinsed with CH_2_Cl_2_ and dried in vacuo.

The cleavage of the peptide from the resin was performed using a mixture of TFA/phenol/H_2_O/thioanisole/EDT/triisopropylsilane (80.5/5/5/2.5/2) for 2 h. After evaporation of the solvent and precipitation in *t*-butylmethylether, the crude peptide was washed 3 times with *t*-butylmethylether and dissolved in acetic acid (6 mL). Then the solution was diluted with water (800 mL) and adjusted to pH 8 employing 25% NH_3_ and vigorous stirring was performed in order to ensure saturation with air for the oxidative cyclization of the cysteine thiol residues. After 2 d, the mixture was lyophilised and purified using preparative RP-HPLC (eluent: CH_3_CN (A); 0.1% HCO_2_H in H_2_O (B) applying a linear gradient 10%–18% (A) in 26 min).

For FProp-C^*^RRETAWAC^*^-OH, two epimers were found (*t*_*r*_ epimer 1: 17.0 min, and epimer 2: 18.4 min). In order to obtain a pure fraction of epimer 2, a second HPLC-separation had to be performed. Peptide purity and identity were assessed by analytical HPLC employing the following eluent system: 3–30% CH_3_CN in 97–70% H_2_O + 0.1% HCO_2_H in 26 min, epimer 1: purity: >99% (*t*_*r*_: 20.2 min), epimer 2: purity: >99% (*t*_*r*_: 21.2 min); ESI-ToF-MS: [M+H]^+^, calculated for C_47_H_72_FN_16_O_14_S_2_: 1167.4839, found: 1167.4831.

#### 2.1.4. Ala-Scan-Peptides

Peptides modified with alanine on the different positions and used for the Ala-scan (see Table 1) have been purchased from Biosynthan (Berlin, Germany). These peptides were analysed by MALDI-TOF and HPLC. Supplied peptides were >95% pure.

### 2.2. [^18^F]labelling of H-C^*^RRETAWAC^*^-OH

For labelling H-C^*^RRETAWAC^*^-OH, (*RS*)-2-[^18^F]fluoropropionate (FProp) was used as a prosthetic group. labelling of the compound was carried out in a semiautomated system, which has been established at the Department of Nuclear Medicine in Innsbruck. Synthesis of the prosthetic group was carried out as described previously [17] and followed the protocols from Guhlke et al. [19]. For the final conjugation step, 4-nitrophenyl-(*RS*)-2-[^18^F]fluoropropionate (100–200 MBq) was coated in a plastic vial and the corresponding peptide (0.5–1.0 mg), dissolved in anhydrous DMSO (200 *μ*L), was added and heated in the presence of 3 eq. KOBt (potassium salt of 1-hydroxybenzotriazole (HOBt)) for 10 min at 70°C. After reaction, the mixture was transferred to a syringe and the vial was washed with DMSO (200 *μ*L) and water (200 *μ*L). Combined solvents were injected onto semipreparative HPLC (Gynkotech 480 High Precision Pump, a Gynkotech SP6V UV detector (Germering, Germany), and a Bioscan radiometric detector with a Multo-High 100 RP 18–5 *μ*m column (250 × 10 mm; CS-Chromatography, Langerwehe, Germany) using the following gradient: 10–50% CH_3_CN/water/0.1% TFA in 30 min; flow rate 5 mL/min), and the corresponding radio peak was collected. The HPLC solvent was removed by evaporation and the title compound [^18^F]FProp-C^*^RRETAWAC^*^-OH was reconstituted in PBS (pH 7.4) for subsequent* in vitro* and* in vivo* experiments.

### 2.3. *In Vitro* Characterisation

#### 2.3.1. Partition Coefficient

[^18^F]FProp-C^*^RRETAWAC^*^-OH (approx. 1 kBq) in PBS (250 *μ*L, pH 7.4) was added to octanol (250 *μ*L) and the mixture was vigorously vortexed for 15 min. Subsequently, aliquots of the aqueous and the octanol layer were collected (each 75 *μ*L) and measured in the gamma counter, and logD values were calculated (*n* = 5).

#### 2.3.2. Protein Binding Assay

The protein binding studies were carried out by incubating [^18^F]FProp-C^*^RRETAWAC^*^-OH (1 MBq/mL) in fresh human serum at 37°C for different time points (30, 60, and 120 min). After incubation, the solution was passed through a spin column (MicroSpin G-50 columns; GE Healthcare, Buckinghamshire, United Kingdom). Protein binding was determined by measuring the activity fixed on the column and the activity in the eluate in the gamma counter. As control [^18^F]FProp-C^*^RRETAWAC^*^-OH was incubated at the same concentration for 60 min in PBS instead of serum.

#### 2.3.3. Stability in Human Serum

For stability studies, [^18^F]FProp-C^*^RRETAWAC^*^-OH (approx. 2 MBq/mL) was incubated in human serum for 30, 60, and 120 min at 37°C. At each time point, aliquots of 100 *μ*L were taken and proteins were precipitated using 150 *μ*L acetonitrile. The suspension was treated for 5 min with ultrasound and subsequently centrifuged. The supernatant was diluted with water and analysed by HPLC.

#### 2.3.4. Determination of *K*_*i*_-Values Using Isolated Receptor Binding Assays

The receptor binding assay was performed following the procedure described previously [20]. In brief, commercially available purified*α*_*v*_ *β*_3_,*α*_5_ *β*_1_, or*α*_*IIb*_ *β*_3_ (Triton X-100 formulation, Chemicon, Millipore) was diluted in coating buffer (25 mM Tris-HCl, 150 mM NaCl, 1 mM CaCl_2_, 0.5 mM MgCl_2_, 1 mM MnCl_2_, pH 7.4), and an aliquot of 100 *μ*L/well, corresponding to 25 ng/well*α*_*v*_ *β*_3_, 100 ng/well*α*_5_ *β*_1_, or 50 ng/well*α*_*IIb*_ *β*_3_, was added to a 96-well microtiter plate (Maxisorb, Nunc, Wiesbaden, Germany) and incubated at 4°C overnight. The cells were incubated for two hours with blocking buffer (200 *μ*L, coating buffer containing 1% (w/v) bovine serum albumin (BSA)) at room temperature. After washing twice with binding buffer (coating buffer with 0.1% (w/v) BSA) the cells were incubated in the presence of the competing ligand (90 *μ*L, 0.5 pM to 500 nM echistatin; 0.05 nM to 50 *μ*M peptide in binding buffer) with [^125^I]echistatin (0.37 kBq/well, 10 *μ*L, 0.05 nM; PerkinElmer, Germany) for 3 h. Afterwards, the cells were washed three times with binding buffer, and bound [^125^I]echistatin was solubilised with warm NaOH (2 M). The radioactivity in the resulting samples was measured by a *γ*-counter. Each data point represents the mean from three cells. All measurements were repeated at least once. *K*_*i*_ values were calculated using the software program GraphPad PRISM (GraphPad Software Inc., CA, USA).

#### 2.3.5. Characterisation of the Murine Tumour Models

(*1) Flow Cytometry*. Cells were washed with cold PBS and incubated with mouse anti-human integrin*α*_5_ *β*_1_ (MAB 1999, Millipore, 1 : 75), mouse anti-human integrin*α*_*v*_ *β*_3_ (MAB1976, clone LM609, Millipore, 1 : 75), or a negative control IgG (DAKO) for 30 minutes at 4°C, followed by incubation with a FITC-labelled goat anti-mouse secondary antibody (1 : 400, 2.5 *μ*L in 1 mL DAKO). After careful washing to remove unbound antibodies, samples were analysed on a FACSCalibur flow cytometer (Becton Dickinson, USA). The analysis was performed by Cell Quest software version 4.0.1 (Becton Dickinson, USA). 

*(2) Immunofluorescent Staining.* Three mice with DU145 tumour xenografts induced as described below were sacrificed and tumour tissue was removed and frozen in liquid nitrogen. Frozen tissue sections were fixed with acetone for 10 minutes, followed by blocking with PBS + 10% goat serum for 20 minutes at room temperature. Then, slides were incubated with a mouse monoclonal antibody against*α*_5_ *β*_1_ integrin (MAB 1999, Millipore, 1 : 50) for 1 hour at 37°C. Negative control sections were stained with an unspecific isotype control (DAKO). After washing, samples were incubated with an Alexa Fluor 555 goat anti-mouse IgG (Molecular Probes, 1 : 800) for 30 minutes at 37°C. After washing, the slides were covered with Vectashield mounting medium (Vector Labs) with DAPI (4′,6-diamidino-2-phenylindole) and analysed with an Axio Imager Z2 microscope (Zeiss, Vienna) equipped with a Pixelink PL-B622-CU camera (Canimpex Enterprises Ltd., Halifax, NS, Canada).

#### 2.3.6. Cell Uptake Studies

M21 (*α*_*v*_ *β*_3_-positive and*α*_5_ *β*_1_-negative), DU154 (*α*_*v*_ *β*_3_-negative and*α*_5_ *β*_1_-positive), and M21L (both receptors negative) cells were maintained in Gibco RPMI 1640 (Life Technologies, Carlsbad, CA) containing 1% glutamine, 10% (v/v) fetal calf serum (FCS), and 1% penicillin/streptomycin/glutamine and grown in tissue culture flasks (Cellstar; Greiner Bio-One, Kremsmuenster, Austria) at 37°C in a humidified atmosphere of 5% CO_2_ (v/v). For cell uptake studies, cells were trypsinised, washed twice with binding buffer (10 mM HEPES (pH 7.4), 150 mM NaCl, 2 mM MnCl_2_, 0.1% BSA), and aliquots of 0.9 mL containing 2 × 10^6^ cells were transferred to Eppendorf tubes. After addition of 50 *μ*L radiotracer (>100,000 cpm), cells were incubated at 37°C for 120 min in triplicate with either 50 *μ*L PBS with 0.5% BSA (unblocked) or 50 *μ*L of 50 *μ*M H-C^*^RRETAWAC^*^-OH in PBS/0.5% BSA (blocked), respectively.

Incubation was stopped by centrifugation (2 min, 1000 rpm); medium was removed and cells were washed twice with 1 mL ice-cold binding buffer. Subsequently, cells were incubated in acid wash buffer (20 mM acetate buffer pH 4.5) for 10 minutes at 37°C. The supernatant was collected (membrane bound radioligand fraction) and the cells were washed with acid wash buffer. Cells were lysed by treatment in 1 N NaOH and receptor bound radioactivity was collected (internalised radioligand fraction). Protein content in the NaOH fraction was determined using spectrophotometric determination with Bradford reagent (Sigma Aldrich, Vienna, Austria). Internalised radioactivity was expressed as percentage of total activity per mg protein.

### 2.4. *In Vivo* Characterisation

All animal experiments were conducted in compliance with the Austrian animal protection laws and with the approval of the Austrian Ministry of Science (BMBWK-66.011/0066-BrGT/2006). Animal studies were performed using BALB/c nude mice (Charles River Laboratories, Sulzfeld, Germany). For the induction of tumour xenografts, human prostate cancer DU145 cells (*α*_*v*_ *β*_3_ negative and*α*_5_ *β*_1_ positive) as well as human melanoma M21 cells (*α*_*v*_ *β*_3_ positive and*α*_5_ *β*_1_ negative) were injected subcutaneously at a concentration of 5 × 10^6^ cells per mouse and allowed to grow until tumours of 0.3–0.6 cm^3^ were visible.

#### 2.4.1. Biodistribution Studies

A group of 10 mice bearing the DU145 tumour xenograft was injected with [^18^F]FProp-C^*^RRETAWAC^*^-OH (approx. 0.5 MBq per animal in 150 *μ*L PBS pH 7,4) into a lateral tail vein. The animals were sacrificed by cervical dislocation 30, 60, and 120 min after injection. Organs (heart, stomach, lung, spleen, liver, pancreas, kidneys, and intestine), tissues (blood, muscle, and femur), and tumours were removed and weighed. Activity concentration in the samples was measured in the gamma counter. Results were expressed as percentage of injected dose per gram of tissue (% ID/g).

For determination of receptor selectivity of the tracer uptake distribution in mice bearing the human melanoma, M21 tumour xenograft as well as blocking with different amounts of H-C^*^RRETAWAC^*^OH (200 *μ*g and 300 *μ*g) was studied. For each group, 4 animals were injected with [^18^F]FProp-C^*^RRETAWAC^*^-OH (approx. 0.5 MBq per animal in 150 *μ*L PBS pH 7.4) intravenously into the lateral tail vein. The animals were sacrificed by cervical dislocation 60 min after injection. Further processing was similar as described above.

## 3. Results

### 3.1. Peptide Synthesis and Radiolabelling

The peptides were assembled on solid support using Fmoc-protocols (a list of all peptides used in the study is found in Table 1). Disulfide-bridge formation of the CRRETAWAC-peptides was carried out using either hydrogen peroxide or oxygen. All peptides have been synthesised in purity higher than 95%. For the reference compound, conjugation of the racemic 2-fluoropropionate resulted in the formation of two epimeres, which could be separated by HPLC and tested for binding affinity separately (see below).

Peptide labelling was carried out using 4-nitrophenyl-(*RS*)-2-[^18^F]fluoropropionate which was synthesised as described previously [17]. For the final conjugation step, the prosthetic group was coated in a plastic vial. It is of utmost importance to avoid any trace of water in this reaction. For the acylation, the peptide, dissolved in anhydrous DMSO, was added and heated for 10 min at 70°C. This procedure allowed radiolabelling of H-C^*^RRETAWAC^*^-OH with radiochemical yields of approx. 2.3%. To improve the radiochemical yield, 3 eq. KOBt was added to the peptide. This optimised procedure allowed [^18^F]labelling of H-C^*^RRETAWAC^*^-OH with radiochemical yields of approx. 55%. After final HPLC preparation, the radiochemical purity was in general >95%. Under the used HPLC conditions, no epimeres could be identified/separated for the radiolabelled derivatives.

### 3.2. *In Vitro* and* In Vivo* Characterisation

#### 3.2.1. Partition Coefficient, Protein Binding, Serum Stability

**Figure 1:**
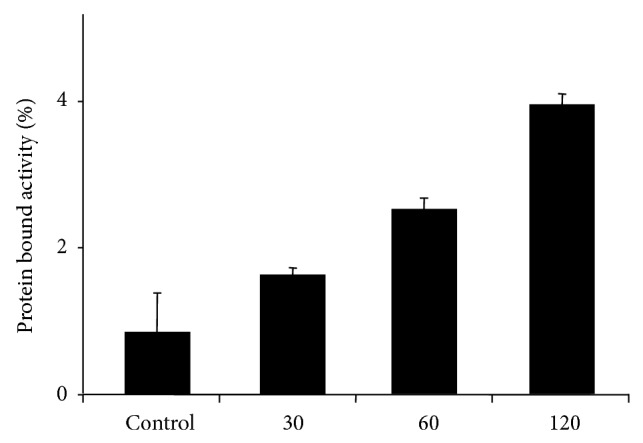
Amount of protein fraction after incubation of [^18^F]FProp-C^*^RRETAWAC^*^-OH in human serum for 30, 60, and 120 min at 37°C. As control, the radiopharmaceutical was incubated in PBS for 60 min 37°C.

**Figure 2:**
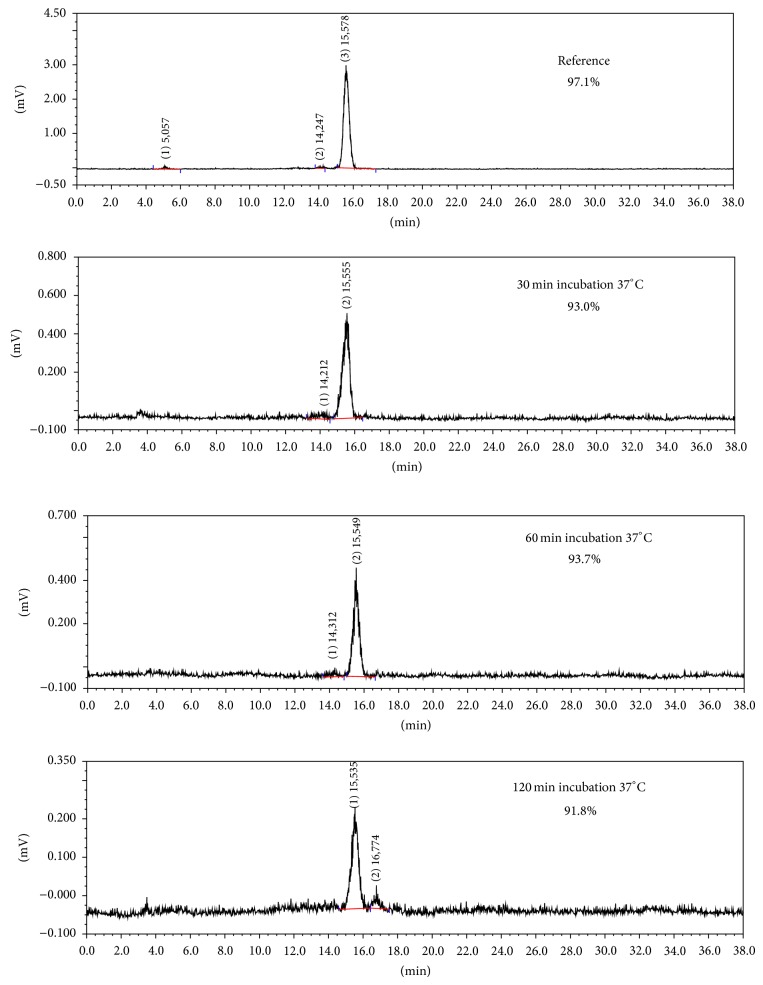
Serum stability of [^18^F]FProp-C^*^RRETAWAC^*^-OH.

Octanol/buffer partition coefficient (logD) of [^18^F]FProp-C^*^RRETAWAC^*^-OH was determined to be −1.9. Protein bound activity of [^18^F]FProp-C^*^RRETAWAC^*^-OH in human serum increased over time but remained below 6% of the total incubated activity after 2 h incubation (Figure 1). The stability of [^18^F]FProp-C^*^RRETAWAC^*^-OH in human serum* in vitro* was excellently high (Figure 2). Over 90% intact tracer was found at all time points during the observation period of 120 min.

#### 3.2.2. Expression Pattern of the Different Cell Lines

**Figure 3:**
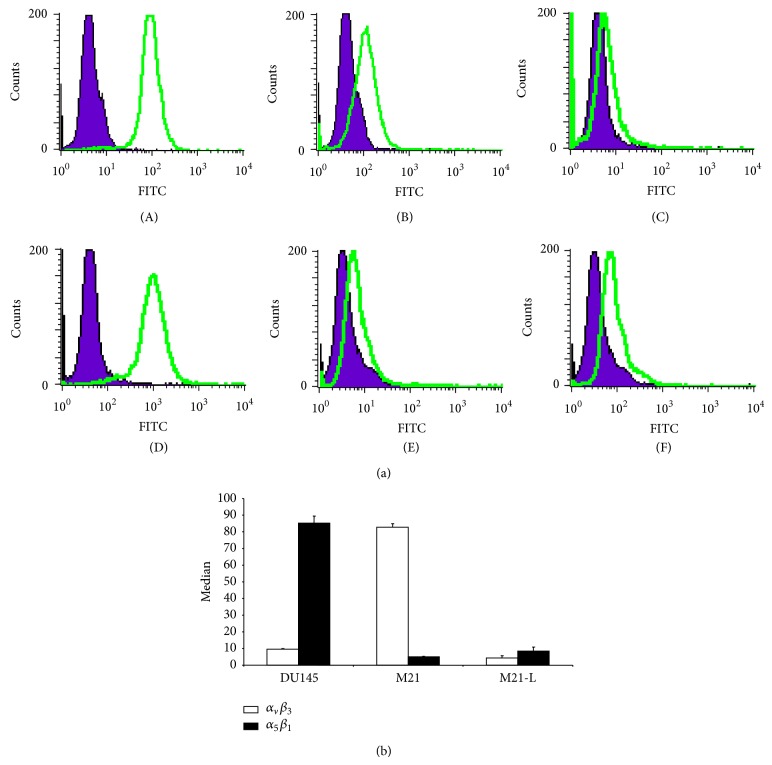
FACS analysis of human prostate carcinoma DU145 as well as human melanoma M21 and M21-L cells. (a): (A) + (B): DU145; (C) + (D): M21; (E) + (F): M21-L; (A), (C), (E): IgG-isotype overlay with*α*_5_ *β*_1_; (B), (D), (F): IgG-isotype overlay with*α*_*v*_ *β*_3_; (b): presentation of the median of the different analysis.

Human prostate cancer (DU145) and human melanoma (M21 and M21-L) cells were analysed regarding*α*_*v*_ *β*_3_ and*α*_5_ *β*_1_ integrin expression (Figure 3; figures show for each integrin and cell line one representative FACS analysis as well as mean values of the median). FACS analysis revealed high*α*_5_ *β*_1_ and low*α*_*v*_ *β*_3_ expression for the DU145 cell line. M21 cells showed a reversed expression pattern. These cells revealed high*α*_*v*_ *β*_3_ and low*α*_5_ *β*_1_ expression. M21-L cells showed not only low*α*_*v*_ *β*_3_ but also low*α*_5_ *β*_1_ expression. Therefore, this cell line was used as negative control cell line for both receptor types.

**Figure 4:**
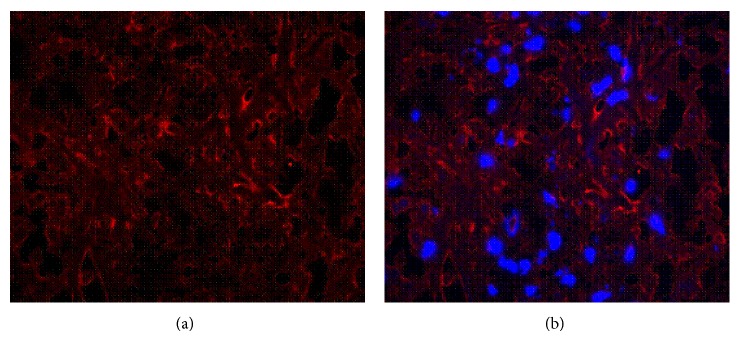
DU145 tumour tissue section stained with a monoclonal anti-*α*_5_ *β*_1_ antibody (a) and costained with DAPI (b) allowing identification of the cell nucleus. Positive*α*_5_ *β*_1_ expression in orange and DAPI staining in blue.

DU145 tumour tissue sections were stained with a monoclonal*α*_5_ *β*_1_ anti-antibody and costained with DAPI allowing identification of the cell nucleus. Fluorescence microscopic analysis of the tumour tissue sections confirmed expression of*α*_5_ *β*_1_ on the murine DU145 tumour model used (Figure 4).

#### 3.2.3. Integrin Binding Data and Cell Uptake Studies

The binding affinities were determined using isolated receptor binding assays where the different integrins (*α*_5_ *β*_1_,*α*_*v*_ *β*_3_, and*α*_*IIb*_ *β*_3_) were immobilised and [^125^I]echistatin was replaced by increasing amounts of the different peptides tested. H-C^*^RRETAWAC^*^-OH and the two isomers of FProp-C^*^RRETAWAC^*^-OH were able to fully suppress the binding of [^125^I]echistatin to the immobilised integrin*α*_5_ *β*_1_ and the binding kinetics followed a classic sigmoid path (corresponding *K*_*i*_ values are found in Table 2). Introduction of the (*RS*)-2-fluoropropionic acid at the N-terminus of the peptide reduced the binding affinity by a factor of approx. 10. The different isomers, which were separated via HPLC, did not show significant differences in the binding to integrin*α*_5_ *β*_1_. Binding affinity to integrins*α*_*v*_ *β*_3_ and*α*_*IIb*_ *β*_3_ was found to be above 50 *μ*M indicating the*α*_5_ *β*_1_-preferring selectivity of the compound.

In a subsequent test series of assays, the influence of the different amino acids on the binding affinity to integrin*α*_5_ *β*_1_ has been determined. Therefore, an Ala-scan was performed and the resulting 5 peptides were compared in their binding capability with the lead structure H-Cys^1^^*^-Arg^2^-Arg^3^-Glu^4^-Thr^5^-Ala^6^-Trp^7^-Ala^8^-Cys^9^^*^-OH. Replacement of Arg^3^ has only minor influence on the binding of the peptide to the integrin*α*_5_ *β*_1_. In contrast, replacement of Arg^2^, Glu^4^, Thr^5^, or Trp^7^ by Ala resulted in a complete loss of the binding affinity indicating that these amino acids are of great importance for binding to integrin*α*_5_ *β*_1_.

**Figure 5:**
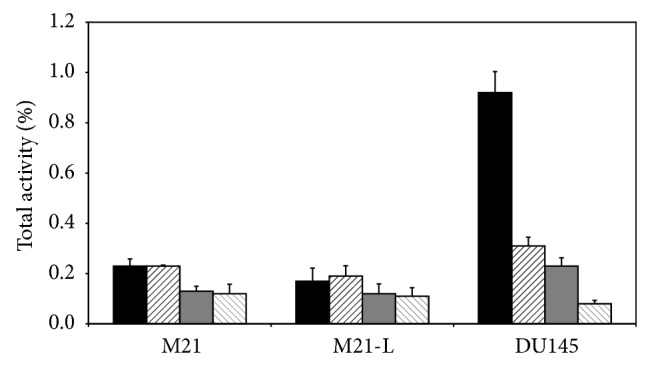
Cell uptake studies of [^18^F]FProp-C^*^RRETAWAC^*^-OH using human melanoma M21 and M21-L as well as human prostate carcinoma DU145 cells. Black: cell bound activity, black striped: cell bound activity blocked, grey: internalised activity, and grey striped: internalised activity blocked.

The corresponding cell uptake studies where cells were incubated for 90 min at 37°C with [^18^F]FProp-C^*^RRETAWAC^*^-OH with or without an excess of the lead structure H-C^*^RRETAWAC^*^-OH showed specific binding and internalisation for the*α*_5_ *β*_1_-expressing DU145 but not for the*α*_*v*_ *β*_3_-expressing M21 cells. In addition, no specific uptake was found in M21-L cells which express neither*α*_5_ *β*_1_ nor*α*_*v*_ *β*_3_ (Figure 5).

#### 3.2.4. Biodistribution and Blocking Studies

Biodistribution and blocking studies were carried out using BALB/c nude mice bearing DU145 tumour xenografts. For additional studies concerning receptor selective tumour uptake, nude mice bearing M21 tumour xenografts were used.

**Figure 6:**
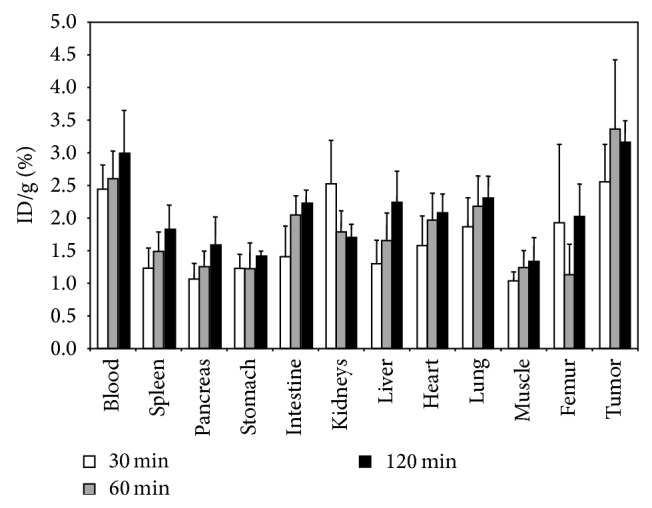
Biodistribution of [^18^F]FProp-C^*^RRETAWAC^*^-OH in nude mice bearing DU145 human prostate carcinoma at different time points postinjection.

Distribution of [^18^F]FProp-C^*^RRETAWAC^*^-OH was determined 30, 60, and 120 min after tracer injection. Data showed highest tracer concentration in the*α*_5_ *β*_1_ positive DU145 tumour tissue for all time points studied with the maximum of 3.4% ID/g at 60 min p.i. (Figure 6). Nevertheless, activity accumulation in blood was only slightly lower and ranged from 2.4% ID/g 30 min p.i. to 3.0% ID/g 120 min p.i. All other organs showed tracer concentration between 1.0% ID/g (muscle 30 min p.i.) and 2.5% (kidneys 30 min p.i.). The kidney was the only organ for which marginal clearance of the tracer was observed. For all other organs including blood there was a constant or even increasing activity concentration found between 30 and 120 min after tracer injection.

**Figure 7:**
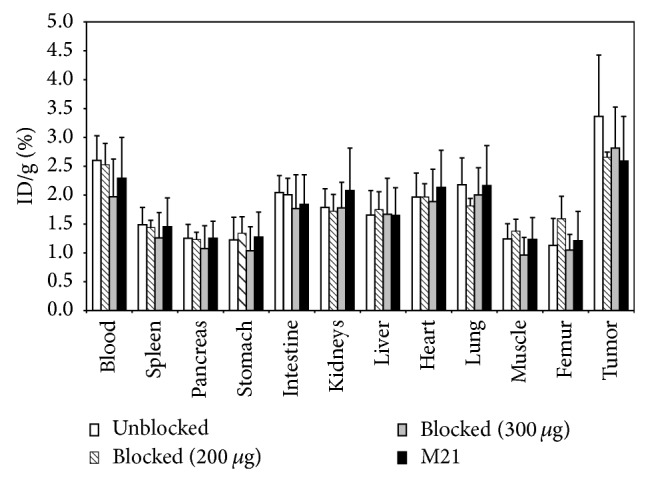
Comparison of the biodistribution data with data from blocking of [^18^F]FProp-C^*^RRETAWAC^*^-OH uptake by coinjection of 200 *μ*g or 300 *μ*g H-C^*^RRETAWAC^*^-OH per mouse in nude mice bearing DU145 human prostate and biodistribution in M21-bearing nude mice (which do not express*α*_5_ *β*_1_).

The initial blocking experiment where 200 *μ*g of H-C^*^RRETAWAC^*^-OH per mouse was coinjected with the tracer showed only low reduction of tracer accumulation in the tumour (Figure 7). However, for all other organs, no reduction or even higher radioactivity concentration was found. To verify if the concentration of the blocking compound was sufficient, an additional experiment using 300 *μ*g H-C^*^RRETAWAC^*^-OH per mouse was carried out. Moreover, uptake in mice bearing M21 tumour xenografts was studied. This tumour does not express the*α*_5_ *β*_1_ integrin (see Figure 3) and was used as a negative control. However, neither the “high dose” blocking nor the experiment with the M21 tumour xenograft bearing mice showed any significant reduction in tracer uptake in the tumour indicating that, if at all, accumulation is only slightly receptor mediated.

## 4. Discussion

Noninvasive imaging of molecular processes during angiogenesis is of utmost importance for both preclinical settings where it may allow new insights in the complex mechanisms as well as for patient care where it may allow more specific planning and controlling of antiangiogenic therapies. One promising target structure family involved in the angiogenic process is the integrins. Until now, most of the studies focused on the integrin*α*_*v*_ *β*_3_. However, other integrins recently came into the focus of interest, including integrin*α*_5_ *β*_1_ [21], which seems to play an even more important role in the angiogenic process than*α*_*v*_ *β*_3_. Based on the nine amino acid sequence H-C^*^RRETAWAC^*^-OH that has been found by phage display techniques [15] and which demonstrated high affinity for the integrin*α*_5_ *β*_1_ in different* in vitro* assays, we aimed at the synthesis and the* in vitro* and* in vivo* characterisation of the [^18^F]labelled derivative [^18^F]FProp-C^*^RRETAWAC^*^-OH to study its applicability as a*α*_5_ *β*_1_-selective PET imaging ligand.

labelling was carried out in analogy to [^18^F]Galacto-RGD [17] using 4-nitrophenyl-(*RS*)-2-[^18^F]fluoropropionate. The initial synthesis resulted in low yields; however, after introduction of the potassium salt of 1-hydroxybenzotriazole (KOBt), which is thought to form an activated ester as reactive intermediate, the yield of the final conjugation step could be increased from 2.3% to approx. 55%. Thus, the peptide could be successfully labelled using this approach. The labelling yields are sufficient even for the use in clinical settings, and this is why this labelling strategy was used for the evaluation of this class of tracer. Anyway, due to the comparably complex labelling strategy, after successful evaluation, alternative labelling strategies have to be developed for an easier translation into a potential clinical routine use.

The binding affinity and receptor selectivity of the modified peptide were controlled using the corresponding isolated immobilised integrins. The selectivity of [^18^F]FProp-C^*^RRETAWAC^*^-OH was comparable to that of the lead structure with high binding affinity that was found for integrin*α*_5_ *β*_1_ and low binding affinity for integrins*α*_*v*_ *β*_3_ and*α*_*IIb*_ *β*_3_. The [^18^F]labelling reaction proceeded with the racemic mixture of 2-[^18^F]fluoropropionic acid ester, thereby resulting in two diastereomeric radiolabelled peptides. However, our* in vitro* binding studies demonstrated no significant difference in the binding affinity for the integrin*α*_5_ *β*_1_ between both diastereomers; thus, for all further experiments, a separation of the radiolabelled diastereomeric products was not carried out. Due to the fact that this peptide sequence resulted from a phage display library screening, it could be expected that the N- and C-terminal end do not have great influence on the binding affinity. In fact, the binding affinity for the N-terminally modified peptide decreased only by a factor <10. In contrast, with exception of Arg^3^, all amino acids of the peptide sequence are important for high affinity binding to the integrin*α*_5_ *β*_1_ with the most dramatic reduction found for Glu^4^ and Trp^7^, as shown by binding studies with the corresponding Ala-modified analogs.

For the preclinical evaluation, human melanoma M21 and M21-L cells as well as human prostate carcinoma DU145 cells have been used. The first cell line has been introduced to control receptor specificity because it is known that M21 cells show high integrin*α*_*v*_ *β*_3_ expression, whereas M21-L cells have low integrin*α*_*v*_ *β*_3_ expression [22]. The latter was chosen because the cell line was used for the evaluation of another*α*_5_ *β*_1_ inhibitor [23], and, therefore, it was assumed that the cells are integrin*α*_5_ *β*_1_-positive. This has been confirmed by our FACS analysis. More importantly, the experiments also showed low*α*_5_ *β*_1_ density for M21 and M21-L cells, whereas, besides high integrin*α*_5_ *β*_1_ expression, low*α*_*v*_ *β*_3_ expression was found for DU145 cells. Thus, by using these three cell lines, we included all necessary controls for the* in vitro* and* in vivo* evaluation and our cell uptake studies demonstrated receptor selective binding and internalisation of [^18^F]FProp-C^*^RRETAWAC^*^-OH only for the DU145, therefore nicely confirming the receptor binding studies on isolated purified integrins.

In contrast, in the* in vivo* biodistribution studies using the DU145 tumour xenograft model, unexpected results were found. At first glance, it was found reasonable that tracer uptake in the tumour was the highest of all organs, but the uptake of the radiopeptide could not be decreased or substantially blocked using increasing amounts of the nonradioactive lead structure H-C^*^RRETAWAC^*^-OH. Moreover, the tracer uptake in the negative control tumour M21 was comparably high. In addition, the radioactivity concentration in the blood was only slightly lower, resulting in unfavourable tumour/blood ratios. Despite the fact that the tracer uptake in tumour was the highest, the other tumour/background ratios were only slightly above one. At the moment, we do not have an intelligible explanation for this finding, especially, having in mind that the amount of blood protein bound radioactivity found by incubating [^18^F]FProp-C^*^RRETAWAC^*^-OH with human serum was low and metabolic stability was high. It is tempting to speculate that the main metabolic degradation of [^18^F]FProp-C^*^RRETAWAC^*^-OH does not occur in blood but in other organs such as the liver. Clearly, additional experiments are needed to study the metabolism of [^18^F]FProp-C^*^RRETAWAC^*^-OH in more detail. However, such analyses of organ homogenates were beyond the scope of the current work.

In summary, despite the high selectivity and affinity for integrin*α*_5_ *β*_1_, selective uptake in receptor-positive cells, and high metabolic stability in human serum* in vitro*, [^18^F]FProp-C^*^RRETAWAC^*^-OH was not suitable to specifically target*α*_5_ *β*_1_-positive tumour cells* in vivo*. Thus, this tracer is not applicable for the noninvasive determination of this receptor and the related angiogenic processes. The Kessler group introduced nonpeptide*α*_5_ *β*_1_ antagonists based on an aza-glycine scaffold and conjugated NODAGA for labelling with ^68^Ga-gallium [14]. In their study, a competitive solid phase integrin binding assay demonstrated high binding affinity and selectivity to integrin*α*_5_ *β*_1_, but in contrast to our data, this compound allowed receptor specific visualisation of the*α*_5_ *β*_1_-positive tumour, which was demonstrated using a murine tumour model of mice bearing an*α*_5_ *β*_1_-positive human colon carcinoma (RKO) on the one flank and the*α*_*v*_ *β*_3_-positive human melanoma (M21) on the other flank.

## 5. Conclusion

In conclusion, [^18^F]FProp-C^*^RRETAWAC^*^-OH could be synthesised straightforward using standard SPPS protocols and labelled in sufficient yields by using 4-nitrophenyl-(*RS*)-2-[^18^F]fluoropropionate. The modified peptide showed comparable selectivity and high affinity to integrin*α*_5_ *β*_1_, which was similar to that of the lead compound, and high stability in serum together with receptor specific uptake* in vitro*. In contrast,* in vivo* studies did not suggest receptor specific binding to integrin*α*_5_ *β*_1_-positive tumours, possibly due to severe metabolic degradation. Further experiments are needed to study the* in vivo* metabolism of this peptide and to develop improved radiopeptide candidates derived from the promising C^*^RRETAWAC^*^-OH sequence that are suitable for PET imaging of*α*_5_ *β*_1_ expression* in vivo*.

## Figures and Tables

**Table 1:** List of the peptides synthesized (asterisks indicate disulfide bridge).

Lead stucture	H-C∗RRETAWAC∗-OH
Reference compound	FProp-C∗RRETAWAC∗-OH
Labelling precursor	H-C∗RRETAWAC∗-OH
Ala-scan	H-C∗ARETAWAC∗-OH
	H-C∗RAETAWAC∗-OH
	H-C∗RRATAWAC∗-OH
	H-C∗RREAAWAC∗-OH
	H-C∗RRETAAAC∗-OH
Labelled compound	[^ 18^F]FProp-C∗RRETAWAC∗-OH

**Table 2:** Binding affinity (*K*_*i*_) of the different compounds tested using the immobilized integrins and ^125^I-echistatin as radioligand (in bold average and standard deviation of the mean are given).

Peptide	*α*_5_ *β*_1_[nM]	*α*_*v*_ *β*_3_[*μ*M]	*α*_*IIb*_ *β*_3_[*μ*M]
H-C∗RRETAWAC∗-OH	10	>50 (*n* = 2)	n.d.
	3.9		
	2.1		
	**5.3 ± 4.1**		

H-C∗ARETAWAC∗-OH	39.000	n.d.	n.d.
	40.000		
	**40.000 ± 1.000**		

H-C∗RAETAWAC∗-OH	17	n.d.	n.d.
	15		
	**16 ± 1 **		

H-C∗RRATAWAC∗-OH	>50.000 (*n* = 2)	n.d.	n.d.

H-C∗RREAAWAC∗-OH	8.000	n.d.	n.d.
	11.000		
	**10.000 ± 2.000 **		

H-C∗RRETAAAC∗-OH	>50.000 (*n* = 2)	n.d.	n.d.

FProp-C∗RRETAWAC∗-OH (1)	41	>50 (*n* = 2)	>50 (*n* = 2)
	47		
	**44 ± 3**		

FProp-C∗RRETAWAC∗-OH (2)	127	>50 (*n* = 2)	>50 (*n* = 2)
	26		
	**77 ± 50**		
